# Hexaaqua­magnesium(II) bis­{[2-(1-phenyl-1*H*-tetra­zol-5-yl)sulfan­yl]acetate}

**DOI:** 10.1107/S1600536810016983

**Published:** 2010-05-15

**Authors:** Chun-Hua Fu, Xiang Zhou, Qing Yu, He-Dong Bian

**Affiliations:** a College of Chemistry and Chemical Engineering, Guangxi Normal University, Guilin, 541004, People’s Republic of China

## Abstract

The asymmetric unit of the title compound, [Mg(H_2_O)_6_](C_9_H_7_N_4_O_2_S)_2_, contains one-half of a [Mg(H_2_O)_6_]^2+^ cation (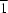 symmetry) and one uncoordinated 2-[(1-phenyl-1*H*-tetra­zol-5-yl)sulfan­yl]acetate anion. The Mg^II^ cation is coordinated by six water mol­ecules, exhibiting a slightly distorted octa­hedral coordination. A two-dimensional network parallel to (001) is formed *via* extensive hydrogen-bonding inter­actions involving the water mol­ecules as donors and the tetra­zole N and carboxyl­ate O atoms of the anion as acceptors. The shortest distance between two adjacent parallel benzene rings is 3.315 (2) Å. The dihedral angle between the benzene ring and the tetra­zole ring is 40.98 (2)°.

## Related literature

For general background, see: He *et al.* (2005 ▶); Yang *et al.* (2008 ▶). For synthetic details, see: D’Amico *et al.* (1957 ▶). For related structures with [Mg(H_2_O)_6_]^2+^ cations, see: Zhang *et al.* (2006 ▶); Zhou *et al.* (2008 ▶).
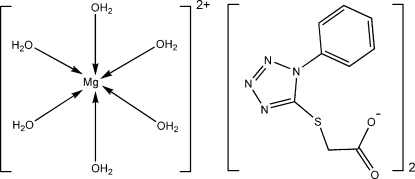


## Experimental

### 

#### Crystal data

[Mg(H_2_O)_6_](C_9_H_7_N_4_O_2_S)_2_
*M* *_r_* = 602.92
Triclinic, 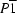

*a* = 6.8380 (14) Å
*b* = 7.5220 (15) Å
*c* = 13.556 (3) Å
α = 92.57 (3)°
β = 99.14 (3)°
γ = 100.07 (3)°
*V* = 675.9 (2) Å^3^
*Z* = 1
Mo *K*α radiation
μ = 0.29 mm^−1^
*T* = 293 K
0.25 × 0.13 × 0.08 mm

#### Data collection

Bruker SMART CCD area-detector diffractometer
Absorption correction: multi-scan (*SADABS*; Bruker, 2001 ▶) *T*_min_ = 0.806, *T*_max_ = 0.931
3914 measured reflections
2347 independent reflections
1867 reflections with *I* > 2σ(*I*)
*R*_int_ = 0.026

#### Refinement

*R*[*F* ^2^ > 2σ(*F* ^2^)] = 0.047
*wR*(*F* ^2^) = 0.122
*S* = 1.02
2347 reflections
202 parameters
H atoms treated by a mixture of independent and constrained refinement
Δρ_max_ = 0.25 e Å^−3^
Δρ_min_ = −0.30 e Å^−3^

### 

Data collection: *SMART* (Bruker, 2001 ▶); cell refinement: *SAINT* (Bruker, 2001 ▶); data reduction: *SAINT*; program(s) used to solve structure: *SHELXS97* (Sheldrick, 2008 ▶); program(s) used to refine structure: *SHELXL97* (Sheldrick, 2008 ▶); molecular graphics: *SHELXTL* (Sheldrick, 2008 ▶); software used to prepare material for publication: *SHELXTL*.

## Supplementary Material

Crystal structure: contains datablocks I, global. DOI: 10.1107/S1600536810016983/wm2320sup1.cif

Structure factors: contains datablocks I. DOI: 10.1107/S1600536810016983/wm2320Isup2.hkl

Additional supplementary materials:  crystallographic information; 3D view; checkCIF report

## Figures and Tables

**Table 1:** Selected bond lengths (Å)

Mg1—O3	2.039 (2)
Mg1—O5	2.061 (2)
Mg1—O4	2.093 (2)

**Table 2:** Hydrogen-bond geometry (Å, °)

*D*—H⋯*A*	*D*—H	H⋯*A*	*D*⋯*A*	*D*—H⋯*A*
O3—H3*A*⋯O2	0.97 (5)	1.77 (6)	2.711 (3)	164 (5)
O3—H3*B*⋯O2^i^	0.75 (4)	2.00 (4)	2.727 (3)	162 (4)
O4—H4*A*⋯O1^ii^	0.77 (4)	2.13 (4)	2.899 (3)	172 (4)
O4—H4*B*⋯N4^iii^	0.92 (4)	2.01 (4)	2.882 (3)	158 (4)
O5—H5*A*⋯N3^iii^	0.84 (4)	2.08 (4)	2.896 (4)	164 (4)
O5—H5*B*⋯O1	0.84 (4)	1.85 (4)	2.682 (3)	172 (3)

Symmetry codes: (i) 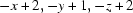; (ii) 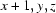; (iii) 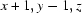.

**Fig. 1.**
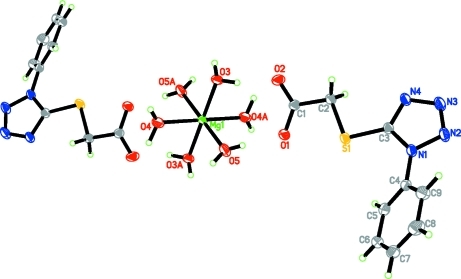
The molecular moieties of (I), showing the atom-numbering scheme. Symmetry code: A -x+2,-y,-z+2. Probability function is drawn at the 50% level.

**Fig. 2.**
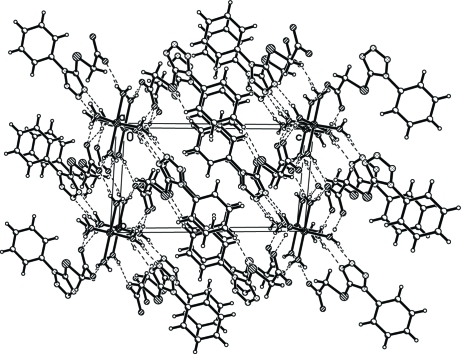
Packing of (I), viewed down the a axis. Hydrogen bonding interactions are shown by dashed lines.

## supplementary crystallographic information

### Comment

The design and synthesis of supramolecular complexes with a high-nuclearity and N-containing carboxylate ligands, especially tetrazole-containing ligands, has been a rapidly growing area of research due to their fascinating structures and interesting physical properties (He *et al.*, 2005). Several transition metal and rare earths metal complexes with similar ligand systems were reported (Yang *et al.*, 2008).

We are interested in the solid-state coordination chemistry of ligands derived from 2-(1-phenyl-1*H*-tetrazol-5-ylthio)acetic acid (HPsta). In order to understand the behavior of alkali earth metals with the HPsta ligand, we prepared the title compound, [Mg(H_2_O)_6_](Psta)_2_, (I), the synthesis and structure of which are reported here.

As shown in Fig. 1, the asymmetric unit of (I) consists of one-half of a [Mg(H_2_O)_6_]^2+^ cation (site symmetry 1) and an uncoordinated 2-(1-phenyl-1*H*-tetrazol-5-ylthio)acetate monoanion. The Mg^II^ atom is coordinated by six water molecules in a slightly distorted octahedral coordination. The corresponding Mg—O distances (Table 1) are in agreement with similar complexes containing the [Mg(H_2_O)]^2+^ cation (Zhang *et al.*, 2006; Zhou *et al.*, 2008). The dihedral angle between the benzene ring and the tetrazole ring is 40.98 (2) °. In the crystal structure, the two Psta groups are involved in a number of intermolecular hydrogen bonds (Table 2) involving the O and N atoms as acceptors and the coordinated water molecules as donor groups (Fig. 2; Table 2), leading to a layer structure extending parallel to (001). In addition, π—π stacking is observed with a shortest distance between two adjacent parallel benzene rings of 3.315 (2) Å.

### Experimental

The ligand 2-(1-phenyl-1*H*-tetrazol-5-ylthio)acetic acid (HPsta) was synthesized according to the literature method (D'Amico *et al.*, 1957). To prepare the title complex, the ligand HPsta (0.4 mmol,0.0944 g) was dissolved in methanol (6 ml) at 348 K and an aqueous solution (4 ml) containing MgCO_3_ (0.0336 g, 0.4 mmol) was added. The resulting solution was stirred at 348 K for 4 h, then cooled to room temperature and filtered. Colorless, prismatic crystals suitable for X-ray diffraction were obtained by slow evaporation over several days, with a yield of 61%. Elemental analysis, found (%):*C*, 35.79; H, 4.38; O, 26.65; N, 18.52; S, 10.66 calc(%): 35.88; H, 4.32; O, 26.58; N, 18.60; S, 10.63.

### Refinement

Water H atoms were located in a difference Fourier map and refined freely. All other H atoms were placed in their calculated positions and refined as riding with U_iso_(H) = 1.2U_eq_(C).

### Crystal data

**Table:**

[Mg(H_2_O)_6_](C_9_H_7_N_4_O_2_S)_2_	*Z* = 1
*M**_r_* = 602.92	*F*(000) = 314.0
Triclinic, *P*1	*D*_x_ = 1.481 Mg m^−^^3^
Hall symbol: -P 1	Mo *K*α radiation, λ = 0.71073 Å
*a* = 6.8380 (14) Å	Cell parameters from 1867 reflections
*b* = 7.5220 (15) Å	θ = 2.0–25.0°
*c* = 13.556 (3) Å	µ = 0.29 mm^−^^1^
α = 92.57 (3)°	*T* = 293 K
β = 99.14 (3)°	Prism, colourless
γ = 100.07 (3)°	0.25 × 0.13 × 0.08 mm
*V* = 675.9 (2) Å^3^	

### Data collection

**Table:**

Bruker SMART CCD area-detector diffractometer	2347 independent reflections
Radiation source: fine-focus sealed tube	1867 reflections with *I* > 2σ(*I*)
graphite	*R*_int_ = 0.026
phi and ω scans	θ_max_ = 25.0°, θ_min_ = 3.1°
Absorption correction: multi-scan (*SADABS*; Bruker, 2001)	*h* = −8→7
*T*_min_ = 0.806, *T*_max_ = 0.931	*k* = −8→8
3914 measured reflections	*l* = −16→16

### Refinement

**Table:**

Refinement on *F*^2^	Primary atom site location: structure-invariant direct methods
Least-squares matrix: full	Secondary atom site location: difference Fourier map
*R*[*F*^2^ > 2σ(*F*^2^)] = 0.047	Hydrogen site location: inferred from neighbouring sites
*wR*(*F*^2^) = 0.122	H atoms treated by a mixture of independent and constrained refinement
*S* = 1.02	*w* = 1/[σ^2^(*F*_o_^2^) + (0.067*P*)^2^ + 0.250*P*] where *P* = (*F*_o_^2^ + 2*F*_c_^2^)/3
2347 reflections	(Δ/σ)_max_ < 0.001
202 parameters	Δρ_max_ = 0.25 e Å^−^^3^
0 restraints	Δρ_min_ = −0.30 e Å^−^^3^

### Special details

**Table:**

Geometry. All esds (except the esd in the dihedral angle between two l.s. planes) are estimated using the full covariance matrix. The cell esds are taken into account individually in the estimation of esds in distances, angles and torsion angles; correlations between esds in cell parameters are only used when they are defined by crystal symmetry. An approximate (isotropic) treatment of cell esds is used for estimating esds involving l.s. planes.

### Fractional atomic coordinates and isotropic or equivalent isotropic displacement parameters (Å^2^)

**Table:**

	*x*	*y*	*z*	*U*_iso_*/*U*_eq_	
Mg1	1.0000	0.0000	1.0000	0.0290 (3)	
C1	0.5668 (4)	0.3487 (4)	0.8626 (2)	0.0316 (6)	
C2	0.4066 (4)	0.4641 (4)	0.8376 (2)	0.0334 (7)	
H2A	0.4655	0.5792	0.8155	0.040*	
H2B	0.3472	0.4874	0.8962	0.040*	
C3	0.0428 (4)	0.4804 (4)	0.72657 (19)	0.0292 (6)	
C4	−0.1942 (4)	0.3060 (4)	0.5768 (2)	0.0321 (7)	
C5	−0.1784 (4)	0.1299 (4)	0.5934 (2)	0.0393 (7)	
H5	−0.1309	0.0985	0.6572	0.047*	
C6	−0.2343 (4)	0.0001 (4)	0.5137 (3)	0.0491 (9)	
H6A	−0.2206	−0.1191	0.5232	0.059*	
C7	−0.3105 (5)	0.0470 (5)	0.4198 (2)	0.0503 (9)	
H7A	−0.3477	−0.0407	0.3664	0.060*	
C8	−0.3314 (5)	0.2215 (5)	0.4051 (2)	0.0526 (9)	
H8A	−0.3861	0.2511	0.3420	0.063*	
C9	−0.2721 (5)	0.3546 (4)	0.4831 (2)	0.0431 (8)	
H9A	−0.2842	0.4740	0.4730	0.052*	
N1	−0.1273 (3)	0.4464 (3)	0.65654 (16)	0.0326 (6)	
N2	−0.2303 (4)	0.5835 (4)	0.66553 (19)	0.0449 (7)	
N3	−0.1245 (4)	0.6943 (4)	0.73682 (19)	0.0465 (7)	
N4	0.0470 (4)	0.6354 (3)	0.77710 (17)	0.0364 (6)	
O1	0.5227 (3)	0.1854 (3)	0.83268 (16)	0.0427 (6)	
O2	0.7301 (3)	0.4261 (3)	0.91271 (18)	0.0550 (7)	
O3	1.0502 (3)	0.2699 (3)	0.97908 (17)	0.0371 (5)	
O4	1.2154 (4)	−0.0513 (3)	0.91404 (18)	0.0453 (6)	
O5	0.7751 (3)	−0.0466 (3)	0.87611 (17)	0.0465 (6)	
S1	0.21753 (11)	0.33953 (10)	0.73900 (5)	0.0384 (2)	
H5B	0.700 (5)	0.027 (5)	0.857 (2)	0.050 (10)*	
H3B	1.122 (5)	0.338 (5)	1.016 (3)	0.048 (11)*	
H5A	0.793 (6)	−0.110 (5)	0.827 (3)	0.069 (12)*	
H4B	1.194 (6)	−0.160 (6)	0.877 (3)	0.088 (14)*	
H4A	1.291 (6)	0.020 (5)	0.893 (3)	0.058 (13)*	
H3A	0.935 (8)	0.313 (7)	0.944 (4)	0.13 (2)*	

### Atomic displacement parameters (Å^2^)

**Table:**

	*U*^11^	*U*^22^	*U*^33^	*U*^12^	*U*^13^	*U*^23^
Mg1	0.0306 (7)	0.0222 (6)	0.0334 (7)	0.0083 (5)	0.0007 (5)	−0.0019 (5)
C1	0.0297 (15)	0.0306 (15)	0.0319 (14)	0.0047 (12)	−0.0013 (12)	0.0008 (12)
C2	0.0324 (16)	0.0308 (14)	0.0343 (15)	0.0082 (12)	−0.0033 (12)	−0.0029 (12)
C3	0.0292 (15)	0.0322 (15)	0.0266 (13)	0.0097 (12)	0.0018 (11)	−0.0003 (12)
C4	0.0255 (15)	0.0358 (16)	0.0339 (15)	0.0065 (12)	0.0024 (11)	−0.0041 (12)
C5	0.0311 (16)	0.0384 (17)	0.0465 (17)	0.0091 (13)	−0.0016 (13)	0.0013 (14)
C6	0.0349 (18)	0.0360 (17)	0.073 (2)	0.0084 (14)	0.0021 (16)	−0.0108 (16)
C7	0.0375 (18)	0.057 (2)	0.053 (2)	0.0069 (15)	0.0059 (15)	−0.0239 (17)
C8	0.051 (2)	0.069 (2)	0.0327 (16)	0.0058 (17)	−0.0001 (14)	−0.0053 (16)
C9	0.0505 (19)	0.0411 (17)	0.0350 (16)	0.0109 (14)	−0.0037 (14)	0.0036 (14)
N1	0.0326 (13)	0.0342 (13)	0.0319 (12)	0.0148 (10)	−0.0002 (10)	−0.0021 (10)
N2	0.0429 (15)	0.0470 (15)	0.0463 (15)	0.0262 (12)	−0.0046 (12)	−0.0083 (13)
N3	0.0496 (16)	0.0457 (16)	0.0458 (15)	0.0250 (13)	−0.0013 (12)	−0.0113 (13)
N4	0.0380 (14)	0.0347 (13)	0.0375 (13)	0.0167 (11)	0.0007 (11)	−0.0051 (11)
O1	0.0356 (12)	0.0297 (11)	0.0581 (13)	0.0117 (9)	−0.0097 (10)	−0.0065 (10)
O2	0.0371 (13)	0.0327 (12)	0.0827 (17)	0.0054 (9)	−0.0239 (12)	−0.0032 (11)
O3	0.0390 (13)	0.0231 (10)	0.0454 (12)	0.0059 (9)	−0.0035 (10)	−0.0018 (10)
O4	0.0471 (15)	0.0331 (12)	0.0552 (14)	−0.0001 (11)	0.0188 (12)	−0.0099 (11)
O5	0.0509 (14)	0.0469 (14)	0.0413 (13)	0.0279 (11)	−0.0094 (10)	−0.0129 (11)
S1	0.0359 (4)	0.0357 (4)	0.0410 (4)	0.0180 (3)	−0.0097 (3)	−0.0103 (3)

### Geometric parameters (Å, °)

**Table:**

Mg1—O3^i^	2.039 (2)	C5—C6	1.383 (4)
Mg1—O3	2.039 (2)	C5—H5	0.9300
Mg1—O5^i^	2.061 (2)	C6—C7	1.382 (5)
Mg1—O5	2.061 (2)	C6—H6A	0.9300
Mg1—O4	2.093 (2)	C7—C8	1.365 (5)
Mg1—O4^i^	2.093 (2)	C7—H7A	0.9300
C1—O2	1.242 (3)	C8—C9	1.383 (4)
C1—O1	1.246 (3)	C8—H8A	0.9300
C1—C2	1.519 (4)	C9—H9A	0.9300
C2—S1	1.803 (3)	N1—N2	1.358 (3)
C2—H2A	0.9700	N2—N3	1.284 (3)
C2—H2B	0.9700	N3—N4	1.367 (3)
C3—N4	1.319 (3)	O3—H3B	0.75 (4)
C3—N1	1.358 (3)	O3—H3A	0.97 (6)
C3—S1	1.725 (3)	O4—H4B	0.91 (5)
C4—C5	1.373 (4)	O4—H4A	0.77 (4)
C4—C9	1.388 (4)	O5—H5B	0.84 (4)
C4—N1	1.438 (3)	O5—H5A	0.84 (4)
			
O3^i^—Mg1—O3	180.000 (1)	C4—C5—H5	120.6
O3^i^—Mg1—O5^i^	90.36 (10)	C6—C5—H5	120.6
O3—Mg1—O5^i^	89.64 (10)	C7—C6—C5	120.1 (3)
O3^i^—Mg1—O5	89.64 (10)	C7—C6—H6A	119.9
O3—Mg1—O5	90.36 (10)	C5—C6—H6A	119.9
O5^i^—Mg1—O5	180.000 (1)	C8—C7—C6	120.4 (3)
O3^i^—Mg1—O4	87.11 (10)	C8—C7—H7A	119.8
O3—Mg1—O4	92.89 (10)	C6—C7—H7A	119.8
O5^i^—Mg1—O4	88.33 (10)	C7—C8—C9	120.6 (3)
O5—Mg1—O4	91.67 (10)	C7—C8—H8A	119.7
O3^i^—Mg1—O4^i^	92.89 (10)	C9—C8—H8A	119.7
O3—Mg1—O4^i^	87.11 (10)	C8—C9—C4	118.5 (3)
O5^i^—Mg1—O4^i^	91.67 (10)	C8—C9—H9A	120.8
O5—Mg1—O4^i^	88.33 (10)	C4—C9—H9A	120.8
O4—Mg1—O4^i^	180.000 (1)	N2—N1—C3	108.3 (2)
O2—C1—O1	125.7 (3)	N2—N1—C4	120.9 (2)
O2—C1—C2	116.6 (2)	C3—N1—C4	130.5 (2)
O1—C1—C2	117.6 (2)	N3—N2—N1	106.2 (2)
C1—C2—S1	107.16 (18)	N2—N3—N4	111.7 (2)
C1—C2—H2A	110.3	C3—N4—N3	105.8 (2)
S1—C2—H2A	110.3	Mg1—O3—H3B	123 (3)
C1—C2—H2B	110.3	Mg1—O3—H3A	115 (3)
S1—C2—H2B	110.3	H3B—O3—H3A	115 (4)
H2A—C2—H2B	108.5	Mg1—O4—H4B	119 (3)
N4—C3—N1	108.1 (2)	Mg1—O4—H4A	127 (3)
N4—C3—S1	129.0 (2)	H4B—O4—H4A	111 (4)
N1—C3—S1	122.93 (19)	Mg1—O5—H5B	125 (2)
C5—C4—C9	121.5 (3)	Mg1—O5—H5A	118 (3)
C5—C4—N1	120.5 (2)	H5B—O5—H5A	110 (3)
C9—C4—N1	118.0 (3)	C3—S1—C2	100.91 (12)
C4—C5—C6	118.9 (3)		
			
O2—C1—C2—S1	164.8 (2)	C5—C4—N1—N2	−142.3 (3)
O1—C1—C2—S1	−16.2 (3)	C9—C4—N1—N2	38.4 (4)
C9—C4—C5—C6	2.6 (4)	C5—C4—N1—C3	44.2 (4)
N1—C4—C5—C6	−176.6 (3)	C9—C4—N1—C3	−135.0 (3)
C4—C5—C6—C7	−2.0 (5)	C3—N1—N2—N3	0.7 (3)
C5—C6—C7—C8	−0.1 (5)	C4—N1—N2—N3	−174.0 (3)
C6—C7—C8—C9	1.6 (5)	N1—N2—N3—N4	−0.5 (4)
C7—C8—C9—C4	−1.0 (5)	N1—C3—N4—N3	0.3 (3)
C5—C4—C9—C8	−1.1 (5)	S1—C3—N4—N3	178.7 (2)
N1—C4—C9—C8	178.1 (3)	N2—N3—N4—C3	0.1 (4)
N4—C3—N1—N2	−0.7 (3)	N4—C3—S1—C2	−0.9 (3)
S1—C3—N1—N2	−179.1 (2)	N1—C3—S1—C2	177.1 (2)
N4—C3—N1—C4	173.4 (3)	C1—C2—S1—C3	176.0 (2)
S1—C3—N1—C4	−5.0 (4)		

Symmetry codes: (i) −*x*+2, −*y*, −*z*+2.

### Hydrogen-bond geometry (Å, °)

**Table:**

*D*—H···*A*	*D*—H	H···*A*	*D*···*A*	*D*—H···*A*
O3—H3A···O2	0.97 (5)	1.77 (6)	2.711 (3)	164 (5)
O3—H3B···O2^ii^	0.75 (4)	2.00 (4)	2.727 (3)	162 (4)
O4—H4A···O1^iii^	0.77 (4)	2.13 (4)	2.899 (3)	172 (4)
O4—H4B···N4^iv^	0.92 (4)	2.01 (4)	2.882 (3)	158 (4)
O5—H5A···N3^iv^	0.84 (4)	2.08 (4)	2.896 (4)	164 (4)
O5—H5B···O1	0.84 (4)	1.85 (4)	2.682 (3)	172 (3)

Symmetry codes:  (ii) −*x*+2, −*y*+1, −*z*+2; (iii) *x*+1, *y*, *z*; (iv) *x*+1, *y*−1, *z*.
